# Supplementary material for: Melatonin-Mediated Colonic Microbiota Metabolite Butyrate Prevents Acute Sleep Deprivation-Induced Colitis in Mice
Source: Int J Mol Sci. 2021 Nov 2;22(21):11894. doi: 10.3390/ijms222111894 (PMC8584377; doi:10.3390/ijms222111894)
Supplement: Supplementary file 1 [file ijms-22-11894-s001.zip › Supplementary Materials.pdf]

Materials and methods

Animal model establishment

Continuous SD of the mice began from 8 am for every day of 3 days using a modified multiple platform water bath as described previously [5]. Eighteen platforms were placed in a water tank. Twelve mice were placed in the water bath. Every mouse in the water bath could move from one platform to another by jumping. The water filled the water bath 4 cm from the base. When the mice had reached the rapid eye movement stage of sleep, which is the paradoxical phase of sleep, muscle atonia could cause the mice to fall into the water. The mice then woke and would try to climb up the platform to avoid being drowned. Throughout the experiments, the water was replaced with clean water in the tank. Moreover, assayed by electroencephalogram (EEG) and electromyogram (EMG) detection (Medusa, Yigebiological, China), mice in the control group could enter slow-wave sleep (Figure S1, Video 1), while sleep deprived mice are active during the whole stage (Figure S2, Video 2). Briefly, MLT (M5250; Sigma, St. Louis, MO, USA) was dissolved in approximately 20 μL of absolute ethanol and diluted with 0.1 mL saline. CON is intraperitoneally injected with vehicle (0.1 mL saline containing 20 μL of absolute ethanol). The SD-treated mice were administered intraperitoneal injections of MLT 0 mg/kg (vehicle, SD, and ABs+SD) and 20 mg/kg (SD+MLT and SD+ABs+MLT) once, 60 min before SD, and a single dose per day at 7:00 am for a total of 3 days. The dose of MLT supplementation were selected based on the published literatures [5]. For substantial depletion of the microbiota, the mice (ABs, SD+ABS, SD+ABs+MLT, and SD+ABs+Butyrate) were provided with drinking water containing 1 g/kg ampicillin (Santa Cruz Biotechnology, Delaware Ave., USA), 100 mg/kg gentamicin (Sigma-Aldrich, St. Louis,

MO, USA), 0.5 g/kg neomycin (Sigma-Aldrich, St. Louis, MO, USA), 0.5 g/kg vancomycin (Hexal, Germany), and 10 mg/kg erythromycin (Sigma-Aldrich, St. Louis, MO, USA) for 10 days (including the acclimatisation and SD periods). For treatment with butyrate, 40 mM sodium butyrate (Sigma-Aldrich, St. Louis, MO, USA) was administered orally to mice by gavage (SD+ABs+Butyrate), 60 min before SD, and a single dose per day at 7:00 am for a total of 3 days.

Faecal material was collected from mice of CON, SD and SD+MLT groups in SD experiment and placed into Eppendorf tubes containing 500 mL of freezing solution (sterile saline solution with 12.5% glycerol) and homogenized. The suspended pellets were then stored at -80 °C until utilized. For FMT, mice were randomized into the following groups: F-CON (antibiotic treatment followed by FMT from CON group), F-SD (antibiotic treatment followed by FMT from SD group), F-SM (antibiotic treatment followed by FMT from SD + MLT group) and F-R (vehicle). Antibiotic mix was administered by oral gavage. The antibiotic treatment was the same as the SD experiment. FMT was carried out via oral gavage with a faecal suspension in a final volume of 0.1 mL. FMT was performed continuous 14 days at per day AM 7:30 after continuous 10 days antibiotic treatment.

All mice were euthanised under anaesthesia using 10% chloral hydrate after the experiment ended at 8:00 am. Their plasma, colon tissue, and colonic contents were harvested. The experiments were repeated twice.

Metabolomics profiling

The mass spectrometer was interfaced with an Agilent 1200 HPLC system. The Q-TOF was calibrated daily using the standard tuning solution from Agilent Technologies. The typical

mass accuracy of the Q-TOF was < 10 ppm. Metabolites were analysed in the positive mode only over a range of 80–1000 m/z using a C18 T3 reverse-phase column from Waters Corporation (Milford, MA, USA) because of the higher number of detected molecular features (i.e., metabolites). MS/MS was generated on the Q-TOF to confirm the identity of the perturbed metabolites. The metabolomics data were submitted to the XCMS Online server (https://xcmsonline.scripps.edu/).

Immunohistochemical staining

The sections were rinsed with 0.01 M PBS (pH 7.4) and incubated with biotinylated goat anti-rabbit IgG (1:200; Sigma, St. Louis, MO, USA) for 2 h at room temperature (23°C ± 2°C). After washing, the tissues were incubated with streptavidin-horseradish peroxidase (1:250, Sigma, St. Louis, MO, USA) for 2 h at room temperature. Immunoreactivity was visualised by incubating the tissue sections in 0.01 M PBS containing 0.05% of 3, 3′-diaminobenzidine tetrahydrochloride (DAB; Sigma, St. Louis, MO, USA) and 0.003% hydrogen peroxide for 10 min in the dark. Control slides without the primary antibody were examined in all cases. Positive cells (presented with yellow-brown staining) were counted in 25 random fields from five cross-sections in each sample. The mean integral optical density of positive cells was then determined using Image-Pro Plus (IPP) image software.

Western blotting

The protein concentration was determined using a bicinchoninic acid (BCA) protein assay kit (Beyotime, P0012). A sample of 20 μg of protein was electrophoresed using 10% sodium dodecyl sulphate-polyacrylamide gel electrophoresis. After electrotransferring the samples onto a polyvinylidene difluoride membrane (Millipore, Billerica, MA, USA), p-P65, p-IκB, and

p-GSK-3β protein were blocked with 1% BSA and other proteins were blocked with 5% skim milk in 1× Tris-buffered saline (TBS) with Tween (TBST) for 2 h at room temperature. The following antibodies were applied: GAPDH, 1:2000; MCT1, 1:500; HDAC3, 1:5000; p-GSK-3β, 1:1000; t-GSK-3β, 1:1000; β-catenin, 1:1000; HIF-1α, 1:2000; p-P65, 1:1000; p-IκB, 1:1000; NLRP3, 1:1000; and Card9, 1:500 (Abcam, Cambridge, MA, USA). Data are expressed as the integral optical density of the bands. The values of the target bands were normalised to the corresponding GAPDH values, and the results were obtained from three repeated experiments.
