# Supplementary figures and images for: Melatonin-Mediated Colonic Microbiota Metabolite Butyrate Prevents Acute Sleep Deprivation-Induced Colitis in Mice
Source: Int J Mol Sci. 2021 Nov 2;22(21):11894. doi: 10.3390/ijms222111894 (PMC8584377; doi:10.3390/ijms222111894)

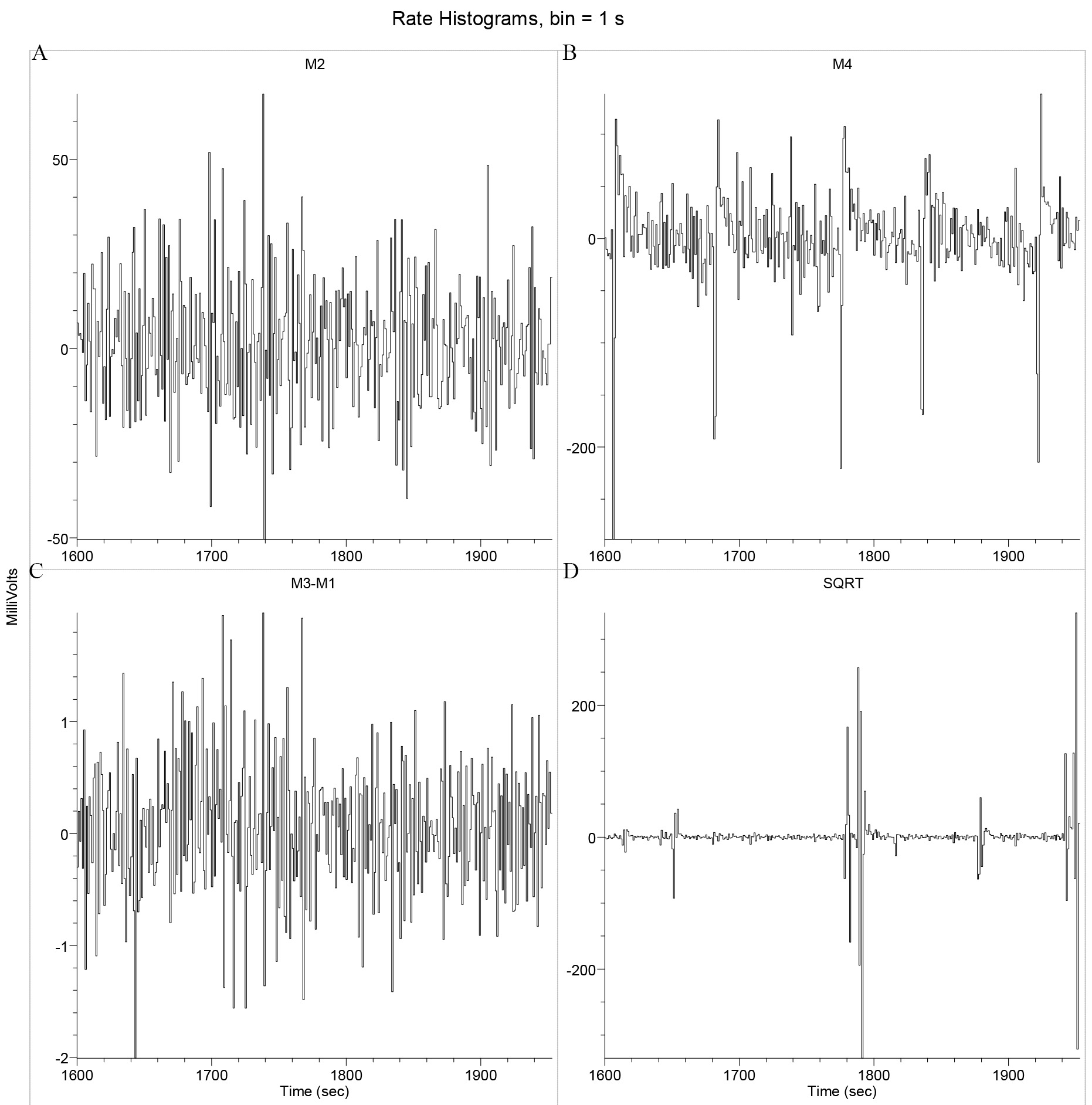

Supplement: Supplementary file 1 [file ijms-22-11894-s001.zip › Figure S1 EEG and EMG detection in mice of control group.jpg]

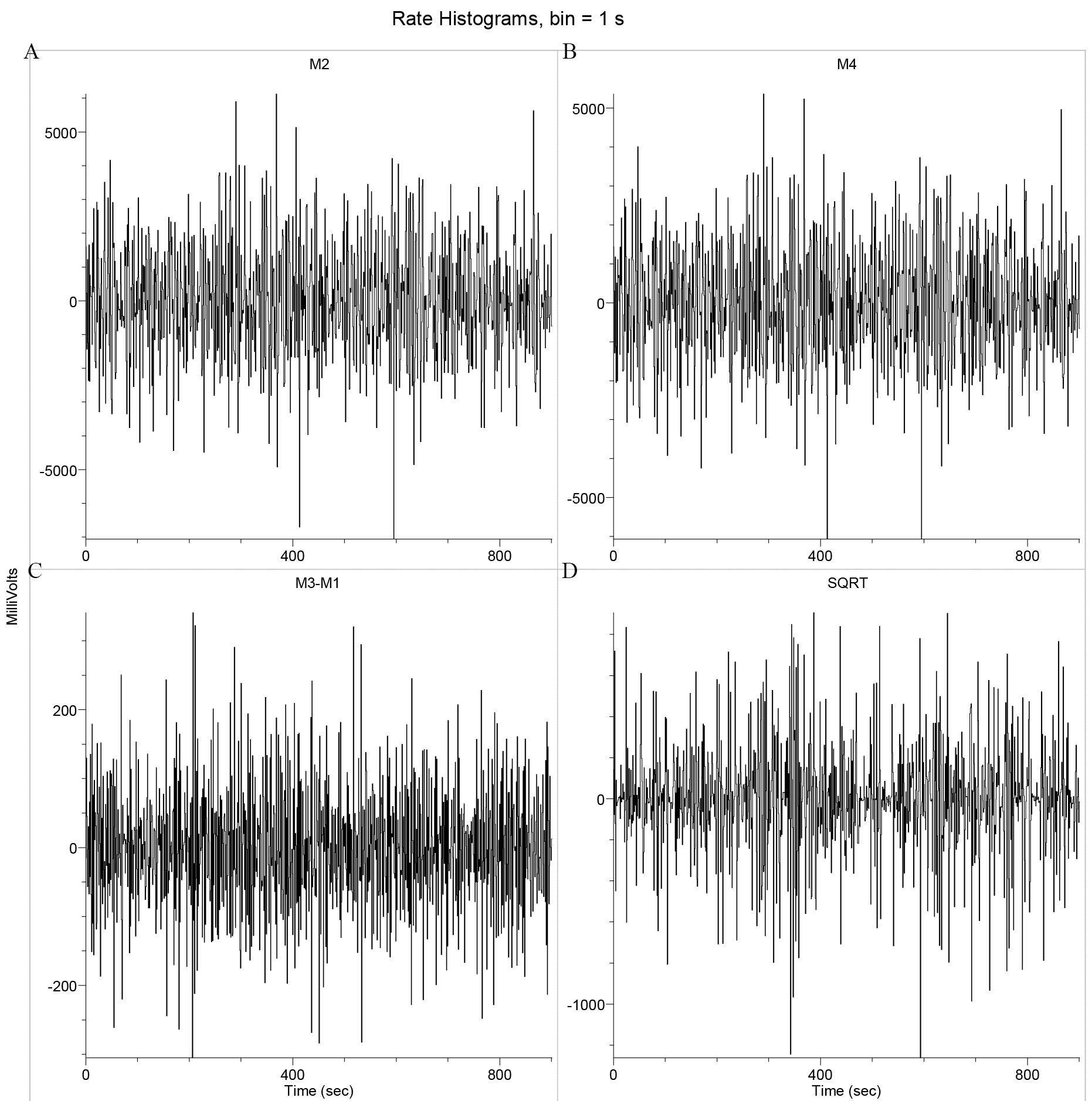

Supplement: Supplementary file 1 [file ijms-22-11894-s001.zip › Figure S2 EEG and EMG detection in mice of SD group.jpg]

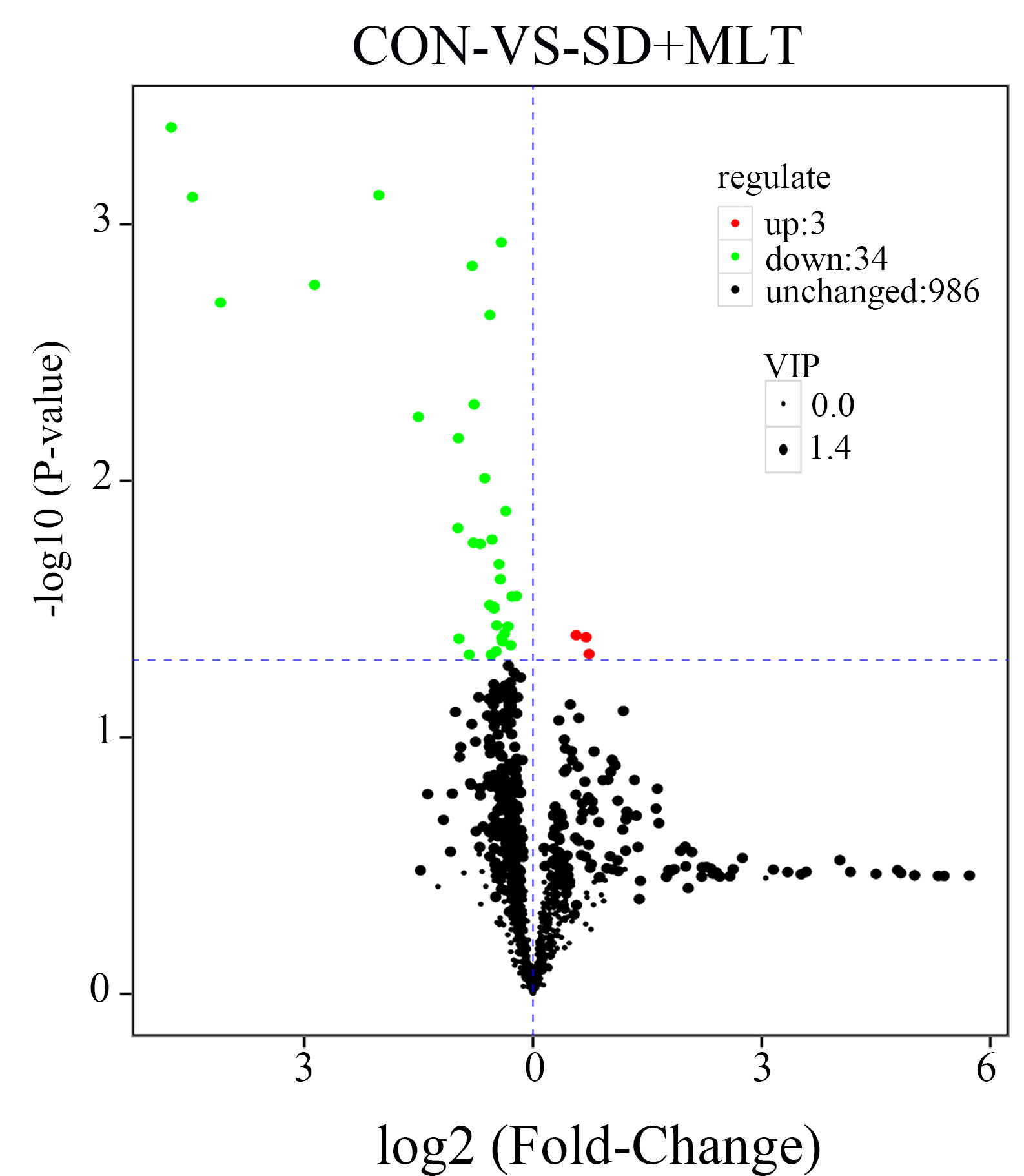

Supplement: Supplementary file 1 [file ijms-22-11894-s001.zip › Figure S3 Volcano plot based on the differential metabolite screening, compared with the CON and SD + MLT groups.jpg]

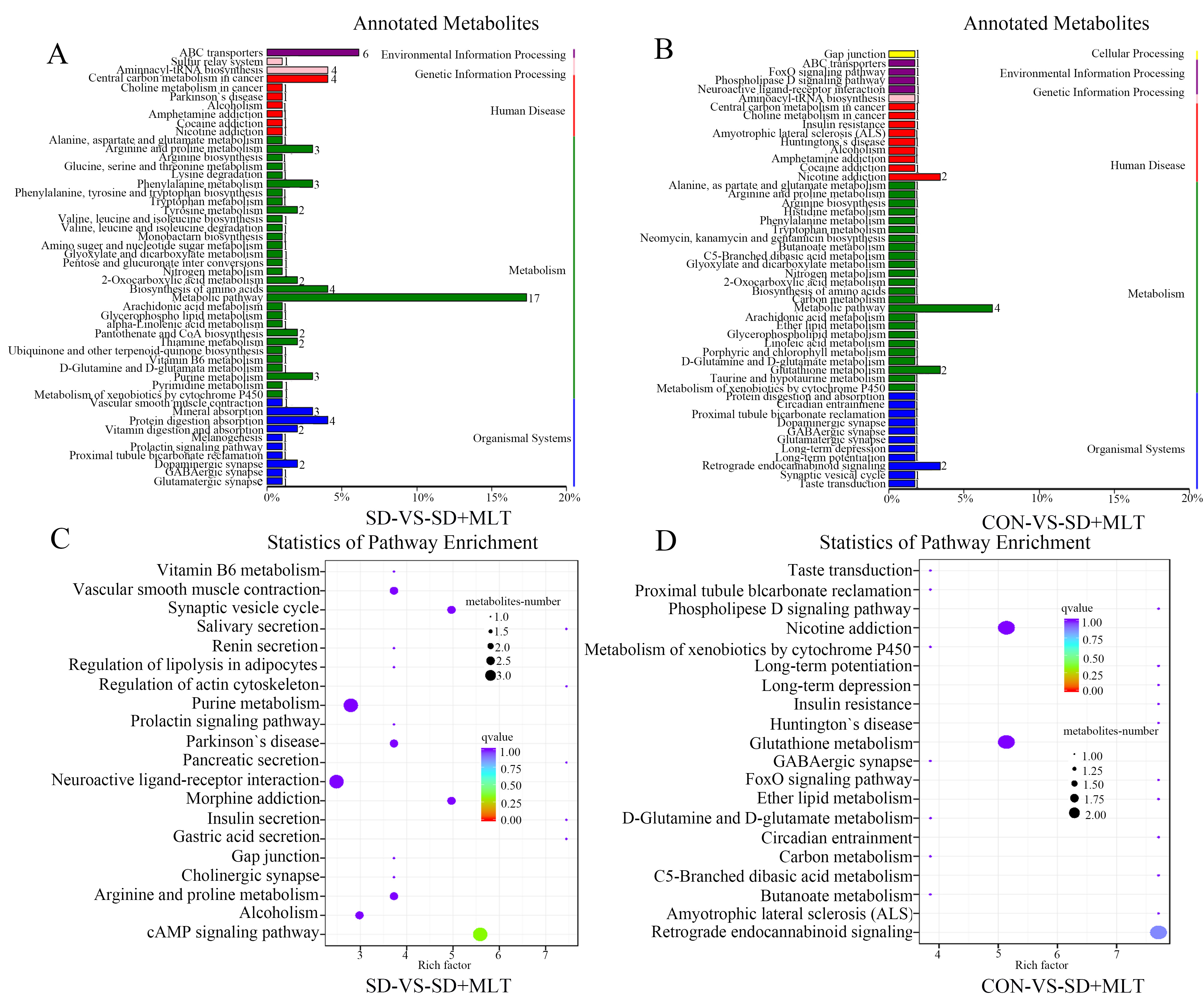

Supplement: Supplementary file 1 [file ijms-22-11894-s001.zip › Figure S4 KEGG analysis.jpg]

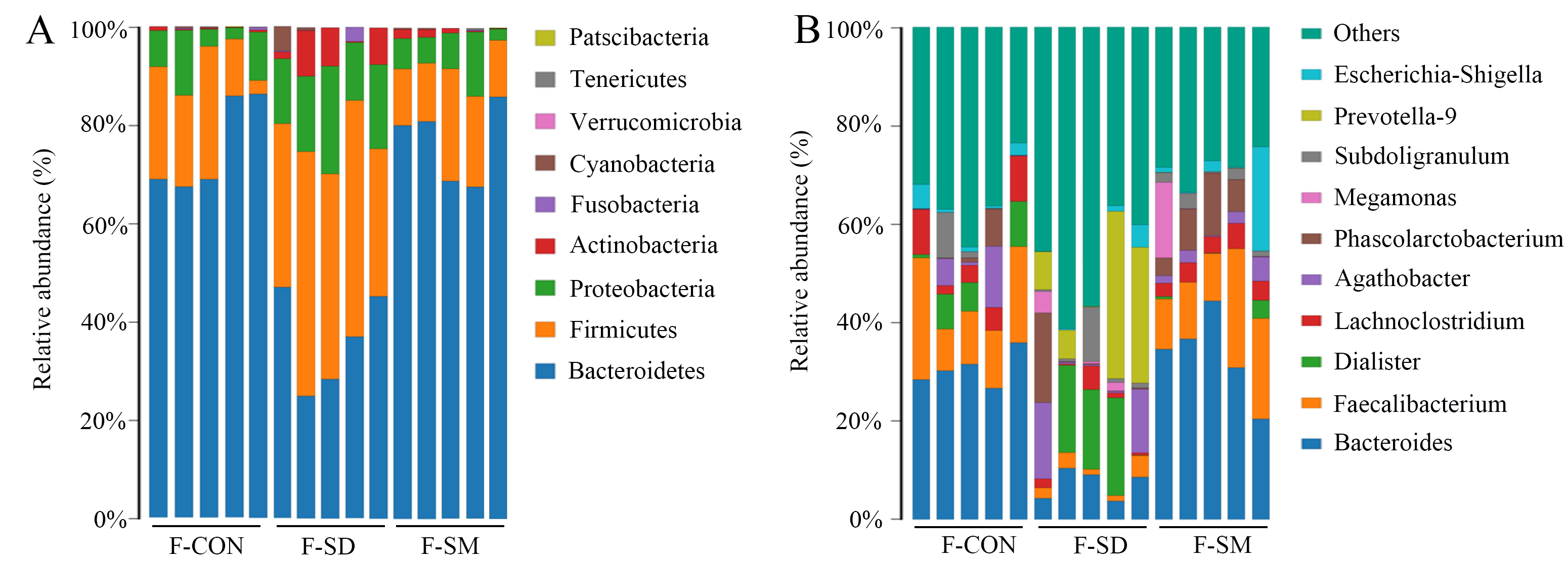

Supplement: Supplementary file 1 [file ijms-22-11894-s001.zip › Figure S5 Composition of the colon microbiota in FMT groups.jpg]
